# Comparing different data sources by examining the associations between surrounding greenspace and children's weight status

**DOI:** 10.1186/s12942-021-00278-w

**Published:** 2021-05-28

**Authors:** Yusheng Zhou, Thomas von Lengerke, Maren Dreier

**Affiliations:** 1 Hannover Medical School, Institute for Epidemiology, Social Medicine and Health Systems Research, Carl-Neuberg Str. 1, 30625 Hannover, Germany; 2 Department of Medical Psychology, Hannover Medical School, Centre for Public Health and Healthcare, Carl-Neuberg Str. 1, 30625 Hannover, Germany

**Keywords:** Greenspace, Children, Pre-schoolers, BMI, Overweight, Spatial source

## Abstract

**Background:**

Studies on the association between surrounding greenspace and being overweight in childhood show inconsistent results, possibly because they differ widely in their definition and measurement of surrounding greenspace. Our aim was to evaluate whether the association of greenspace with being overweight depends on the measurement of greenspace in different data sources.

**Methods:**

Based on data from the school entry examinations of 22,678 children in the city of Hannover, Germany, from 2010 to 14, the association between greenspace availability and overweight was examined. Three different sources of greenspace availability were derived for a set of 51 areas of the city: The Normalized Difference Vegetation Index (NDVI), the OpenStreetMap (OSM) dataset, and the European Urban Atlas (UA) dataset. Agreement between the indicators on the quantity of greenspace coverage was compared. The association with children's BMI z-score, including potential interaction terms, was assessed using multilevel regression analysis.

**Results:**

Greenspace availability per district area derived by NDVI was on average 42%, by OSM 29% and UA 22%, with OSM and UA being strongly correlated. Only the greenspace availability derived by NDVI showed an association with children's BMI z-score: The higher the greenspace availability was, the lower the BMI. The trend of association was higher for boys and migrant children than for girls and non-migrants and was restricted to the highest levels of greenspace availability.

**Conclusions:**

Associations of greenspace with children's weight status depend on the greenspace measurement chosen. Surrounding greenspace was measured more comprehensively by NDVI. Data sources based on land use categories such as UA and OSM may be less suitable to reflect surrounding greenspace relevant for health outcomes. Potential mechanisms warrant further analysis and investigation.

## Introduction

Childhood is a sensitive developmental period in which children acquire more sophistication in interactions determined by a combination of physical, social, family, and built environmental factors. Scholarly interest in greenspace has increased in response to evidence that environmental benefits on children [1, 2]. It has been suggested that exposure to greenspace can contribute to improving public health by multiple pathways, including socio-behaviour (e.g. stress and physical activity) and regulating ecosystem services (e.g. heat reduction) [3, 4]. A shred of growing evidence demonstrates that the rate of overweight is higher among children living in areas lacking sufficient greenspace exposure [5–7]. A study shows that moving to greener neighbourhoods, as measured by density of street trees around home, was associated with lower obesity prevalence [8]. Another study showed that the presence of parks and playgrounds was inversely associated with Body mass index (BMI) [9]. In some studies, using area-level land use data, a null association with BMI was reported [10–12]. The mixed results observed for the association could be due to the differing socioeconomic positions of individuals and the various measurements applied.

As mentioned above, different indices of greenspaces have been used to assess exposures to greenspace in previous epidemiological studies, including the access to greenspace, numbers of greenspace facilities, and the surrounding greenspace (i.e., the presence or absence of greenspace close to participants' residences) [13]. Most studies have introduced a size threshold to represent surrounding greenspace, which refers to the greenspace availability [13, 14]. A UK study assessed the association between physical activity and greenspace availability by measuring the size of the greenspace area [15]. The study emphasized that the size of the greenspace not only matters in terms of the impact on the level of use (quantity) but also because it affects the type of activities (quality) people perform in the area. The larger the greenspace is, the more diverse its flora and the higher its ecological quality are likely to be, which could eventually affect the health and quality of life of populations in the vicinity [16].

Greenspace availability can be formulated differently depending on the data source. Therefore, selecting an appropriate greenspace data source would determine policy intervention, facilitate intercity comparisons, and assess the public health effect. One critical challenge affecting greenspace-related research is the lack of a consistent and strict definition and means of measurement. A range of different geographic datasets are available for assessing greenspace. Some are based on utilization of Landsat satellite images, while others are based on field inventories and mapping. For local-level analysis, such as in one city, local records of greenspace are often preferred because of their high resolution and accuracy [17]. In various European countries, these datasets are prepared by local geographic surveying agencies and constantly updated [18, 19]. However, in many cities, local databases are either not available or not accurate enough. Researchers, in this case, may be obliged or prefer to use more general databases, such as the Urban Atlas (UA) which provides data for European countries [20] or OpenStreetMap (OSM) which provides global data [21]. Generally, the choice of a greenspace dataset depends on the specific needs of the researcher and the availability of the data set. However, poor data availability or inadequate understanding of the current available data may pose a major challenge to analysing greenspace.

In summary, this study's general aim is to determine the associations between surrounding greenspace and children's weight status by applying three different data sources to assess greenspace availability as a built environment indicator. To address this issue, the present study is guided by the following research questions: (1) To what extent do greenspace availability derived from different data sources agree with respect to the surrounding greenspace in the same area? (2) Does the association between greenspace and children's weight status differ across different data sources? (3) Are the associations modified by other individual or social characteristics?

## Methods

### Study area and participants

A cross-sectional study was conducted to explore associations between the degree of greenspace availability and children's body weight. The study was carried out in 51 areas according to the administrative boundaries of the city of Hannover, the capital of Lower Saxony, Germany. The 51 areas (204 km^2^ in total) had a mean area of 4.16 km^2^ ranging from 0.73 to 13.89 km^2^. Data on children's body weight were collected from the school entry examinations conducted annually during school entry registration and through the standardized examination programme "SOPHIA" ("Sozialpädiatrisches Programm Hannover—Jugendärztliche Aufgaben"—http://www.sophia-online.org). The total dataset encompassed individual data on physical and mental health status, personal characteristics, and family socioeconomic status (SES). In this study, we used data from a five-year period from 2010 to 2014, including information on 22,678 children aged 4 to 8 years old.

### Greenspace indicator and data sources

In this study, the greenspace availability in the locating area was used as the indicator to assess surrounding greenspace. It is measured by the proportion of land dedicated to greenspace per administrative unit area defined by local government. Spatial analyses were performed using the open-source geographic information system QGIS (QGIS Development Team, 2014), version QGIS 3.4.5 LTR.

In this analysis of greenspaces in the city of Hannover, the following datasets were selected, adjusting the greenspace categories to make them comparable:

- Landsat satellite images (2013–2014)
- Open Street Map (OSM) (2017)
- Urban Atlas (UA) (2012)

During the preliminary analysis, a local dataset from the Department of Environment and Urban Greenery of Hannover (Fachbereich Umwelt und Stadtgrün) was omitted. This is because the categories of greenspace were not fully interpreted (state forests, greenspace in residential areas with multi-storey housing, private parks and sports facilities were not recognized), which leads to limited accuracy in terms of the greenspace availability.

First, we used the normalized difference vegetation index (NDVI) to assess the greenspace availability in Hannover city [22]. The NDVI data was derived from Landsat 5 Thematic Mapper data at 30 m × 30 m resolution. Its values range from − 1 to 1, with higher values indicating a higher proportion of greenness. Two maps of Hannover city were created using cloud-free images, one from 2013, and another from 2014. From these maps, we calculated an average NDVI as a percentage for each area.

The second source refers to the land use classification by OSM. OSM is a community driven collaborative mapping project that involves contributors from all over the world in the creation of a free, global geospatial database [23]. Countless users are constantly updating open and publicly available resources for the OSM project, although with different degrees of accuracy for different timeframe and places [23]. This fact must be taken into account when processing OSM data together with other spatial data captured. Here, we chose the versions from the end of 2019, which were the most updated versions available when we started our analysis. All categories of the land-use feature classes corresponding to greenspaces were identified from previous studies (Table 1).

**Table 1:** Characteristics of the greenspace data sources

	NDVI*	OSM*	UA*
Type of measurements	Biophysical variables in vegetation	Data of categorical land uses	Data of categorical land uses
Type of data	Primary data	Primary data	Secondary data
Key characteristics	Using Landsat image to calculate the visible and near-infrared light reflected by vegetation. The result refers to the NDVI	A project which allows the community of Internet users to continuously update open and publicly available resources	Land cover data based on satellite imagery
Data availability	Worldwide provided	Worldwide provided, although with different degrees of accuracy	Data available for major urban cities in the European area
Time scale	Depends on the Landsat satellites	Continuously update	Data prepared periodically, currently from 2006, 2012 and 2018
Responsible agency	Landsat images available from the United States Geological Survey (USGS)	Database maintained by the OpenStreetMap Foundation	Project coordinated by the European Environment Agency (EEA)
Greenspace categories identified	Greenspace were identified with the use of supervised classification based on the representative samples for the different green space types in the digital image	– Allotments – Cemetery – Farmland/ farmyard – Forest/wood – Garden – Grassland – Greenfield – Greenhouse horticulture – Meadow – Nature reserve – Orchard – Park – Plant nursery – Scrub – Trees – Village green – Wetland	– Green urban areas (Code 14,100) – Arable land (annual crops) (Code 21,000) – Permanent crops (Code 22,000) – Pastures (Code 23,000) -Complex and mixed cultivation (Code 24,000) – Forests (Code 30,000) – Herbaceous vegetation associations (Code 32,000) – Wetland (Code 40,000)

^*^NDVI: Measurement of calculating the Normalized Difference Vegetation Index (NDVI) by analysising Landsat satellite images; OSM: the OpenStreetMap dataset; UA: the European Urban Atlas dataset

For the third source of greenspace, we used the Urban Atlas data recorded in 2012 by the European Environment Agency. The Urban Atlas provides detailed vector data on land cover and land use for numerous city regions in Europe [24]. Such data are available at a scale of 1:10 000 for 2006, 2012 and 2018 with a largely standard nomenclature (max. 28 classes). For the latter reference year, the Urban Atlas has been available since 2018 for 788 Functional Urban Areas of Europe as spatial and statistical data [24]. The Urban Atlas is derived from remote sensing (e.g., SPOT satellite images) and contains overlap-free geo-objects (polygons) suitable for GIS processing. Here, we chose UA-2012 to match the year of spatial data with the school examination data (2010–2014). Urban Atlas data on surrounding greenspaces were identified based on the relevant mapping guides' descriptions (Table 1).

### Health outcome and covariates

Objectively standardized measured height and weight were used to calculate the body mass index (BMI) z-score for each subject. The BMI z-score was calculated using the national weight status reference [24]. Weight status was categorized into: normal weight (BMI < 90th percentile), pre-obesity (90th percentile ≤ BMI < 97th percentile), and obesity (BMI ≥ 97th percentile). Categorization as overweight in this study refers to the status including both pre-obesity and obesity (BMI ≥ 90th percentile).

Multiple factors contribute to childhood overweight disparities including cultural, family and socioeconomic status. In this study, demographic and socioeconomic characteristics of children were collected by structured interviews with children and their parents using a standardized questionnaire. Demographic variables included migration background (self-identified and categorized as migrants vs. non-migrants), family structure (nuclear family vs. others) and number of siblings (one or no siblings vs. two or more siblings). Socioeconomic inequalities among parents may be partly mediated by their health behaviours which eventually reflected among their children. Parents’ education is frequently used as an indicator of SES in surveys among children. Also, education may be considered as one of the markers representing childhood social environment, and show differences in awareness of health issues [25]. Here in the school entry examination, parental education status was the only available SES-indicator which was classified into three educational classes (lower, middle, and higher) based on parents' primary qualification and professional education. The birth weight was also included (high: > 4000 g, normal: 2500–4000 g, and low: < 2500 g). In addition, length of child day care participation was included (as at least three years vs. less than three years) to evaluate the influence of preschool childcare services.

On the area level, we selected three sociodemographic characteristics provided by the Statistics Office of State Capital Hannover which publishes the city’s structural data annually [26]: the proportion of residents with migration background in each area, the area’s unemployment rate, and the residential density (i.e., residents per hectare). Data from 2010 to 2014 were selected to match the school entry examination data.

### Data analysis

To address research question 1, we compared the values of greenspace availability for each area using scatter plots. We explored differences in the mean agreement between the measurements of the greenspace data sources. Correlations between two of the three data sources were determined using the Pearson correlation coefficient r. The spatial patterns of surrounding greenspace were then mapped separately using GIS software.

To answer research questions 2 and 3, multilevel regression analysis was applied to explore the associations between surrounding greenspace and the BMI z-score of the children. First, an unconditional model with no predictors was estimated to assess the intraclass correlation of the BMI z-score. Then, all individual-level characteristics (i.e., sex and migration background) and area-level sociodemographic factors (i.e., unemployment rates) were added as fixed effects. All the variables were tested for multicollinearity through bivariate correlation and variance inflation factor (VIF). Consequently, one of the area-level indicators (the proportion of residents with migration background in the area) was dropped from the model. To examine the unique contribution of the three greenspace variables (NDVI, UA and OSM), three different models were calculated (Models 1a, 1b, and 1c). To deal with spatial autocorrelation, we applied the Moran’s I test by including x- and y- coordinates of the unit centroids of each area. As in the analysis, these coordinates did not show to be significant; as a result, they were excluded from the final models. To study whether the changes in children's BMI z-score varied across different sociodemographic subgroups, the data were further analysed as follows: an interaction between greenspace availability and sex, migration background and parental education level was added to the models. A statistically significant interaction term between these factors would suggest that the association between greenspace and children's weight status has a different magnitude in the different categories of that variable (Models 2a, 2b, and 2c).

To study the shape of the association of greenspace availability with being overweight, spline regression models were applied. First, a linear term (1 degree of freedom) was specified. Then, we specified natural splines with 3, 4 and 5 degrees of freedom (df = 2 were too small and therefore excluded from the analysis [27]. To test whether the goodness-of-fit of the spline models was significantly better than that of the linear model, the likelihood ratio test was applied. The plots were presented using the probability of being overweight. Greenspace availability is shown on the x-axis of the plots. To visualize the influence of the interaction terms (sex, migration background and parental education level) on the association, we also performed stratified analyses by these factors in Fig. 3.

P-values < 0.05 were considered statistically significant. All analyses were performed using IBM SPSS Statistics for Windows software, version 25.0 (IBM Corp., Armonk, NY, USA) and R 4.0.3 (R Foundation for Statistical Computing, Vienna, Austria).

## Results

Table 2 shows the socioeconomic, demographic and built environment characteristics of the complete sample. According to the statistics, approximately half of the children were girls (48.5%) and had a migration background (49.4%), and nearly one in ten was overweight (9.7%).

**Table 2:** Characteristics of the study population and the area (data from school entrance examination, city of Hannover, 2010–2014, n = 22,678)

	N (%)	Mean	Standard Deviation	Minimum	Maximum
Dependent variables					
Body mass index percentile	50.1		27.9	0	99.8
Overweight* (> 90th percentile)	9.70%				
Obese > 97th percentile	4.10%				
Individual level factors					
Age			6	0.4	4
Sex					
Boys	51.50%				
Girls	48.50%				
Migration background					
Yes	49.40%				
No	50.60%				
Family structure					
Single parent/ Blended family	2.80%				
Nuclear family	97.20%				
Siblings					
≥ 2 siblings	30.50%				
< 2 siblings	69.50%				
Child day care participation					
< 3 years	18.70%				
≥ 3 years	81.30%				
Parental educational level					
Higher	36.90%				
Middle	26.20%				
Lower	36.90%				
Birth weight					
High (> 4000 g)	10.60%				
Normal (2500–4000 g)	80.10%				
Low (< 2500 g)	6.60%				
Area level factors					
Unemployment rate		8.70%	3.20%	1.90%	16.10%
Proportion of residents with migration background in the area		25.60%	9.50%	6.50%	50.30%
Residential density (residents per hectare)		32.9	32.9	1	168
Greenspace availability indices (Area level)					
Greenspace availibility by NDVI		41.70%	20.00%	0.90%	87.10%
Greenspace availibility by UA		22.30%	19.10%	0.10%	81.50%
Greenspace availibility by OSM		29.80%	16.30%	0.90%	88.90%

^*^ Overweight refers to the status including both pre-obesity and obesity

^*^NDVI: Measurement of calculating the Normalized Difference Vegetation Index (NDVI) by analysing Landsat satellite images; OSM: the OpenStreetMap dataset; UA: the European Urban Atlas dataset

**Fig. 1:**
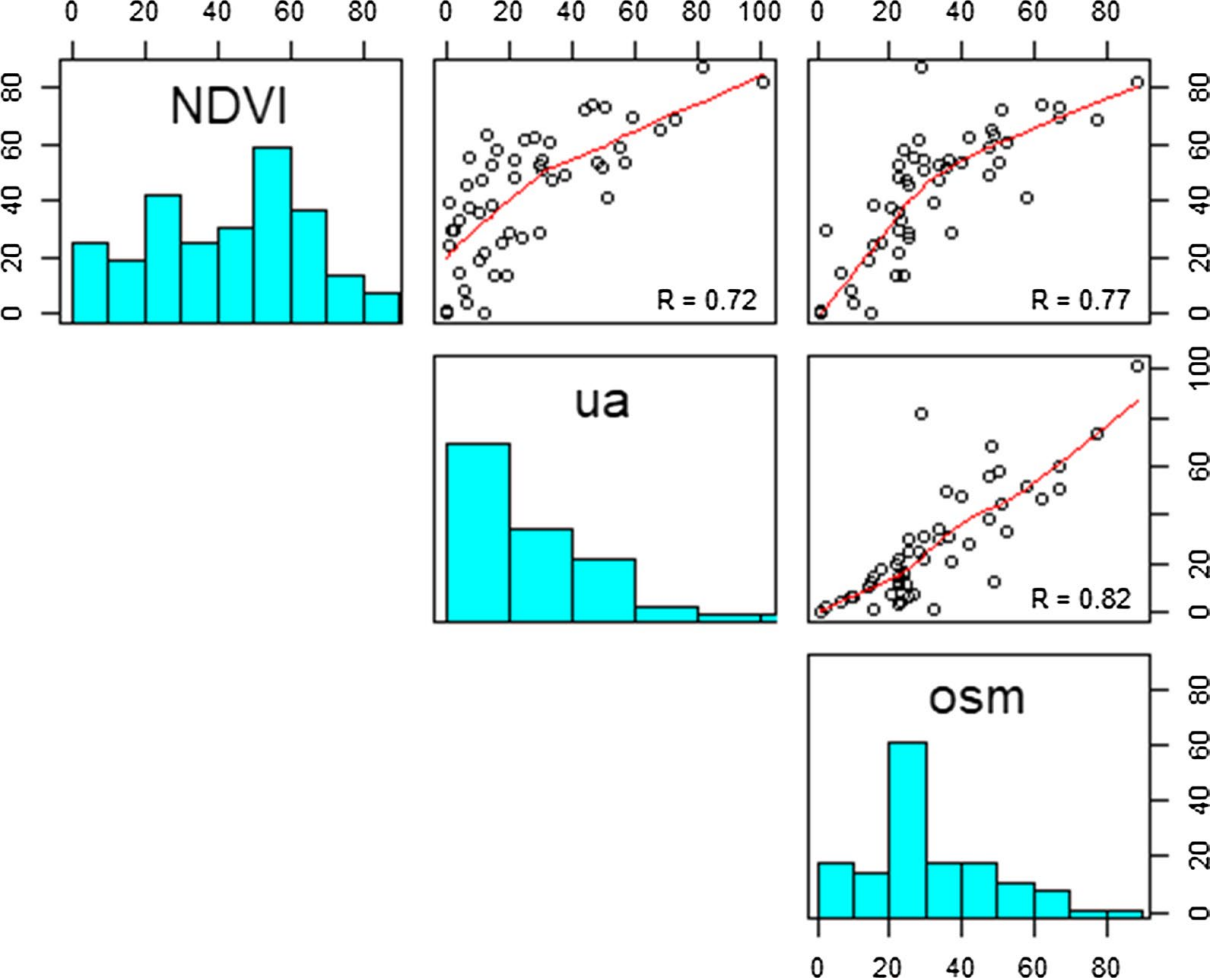
Correlation matrix of the greenspace availabilities from each data sources^$^. ^$^NDVI: Measurement of calculating the Normalized Difference Vegetation Index (NDVI) by analysing Landsat satellite images; OSM: the OpenStreetMap dataset; UA: the European Urban Atlas dataset

**Fig. 2:**
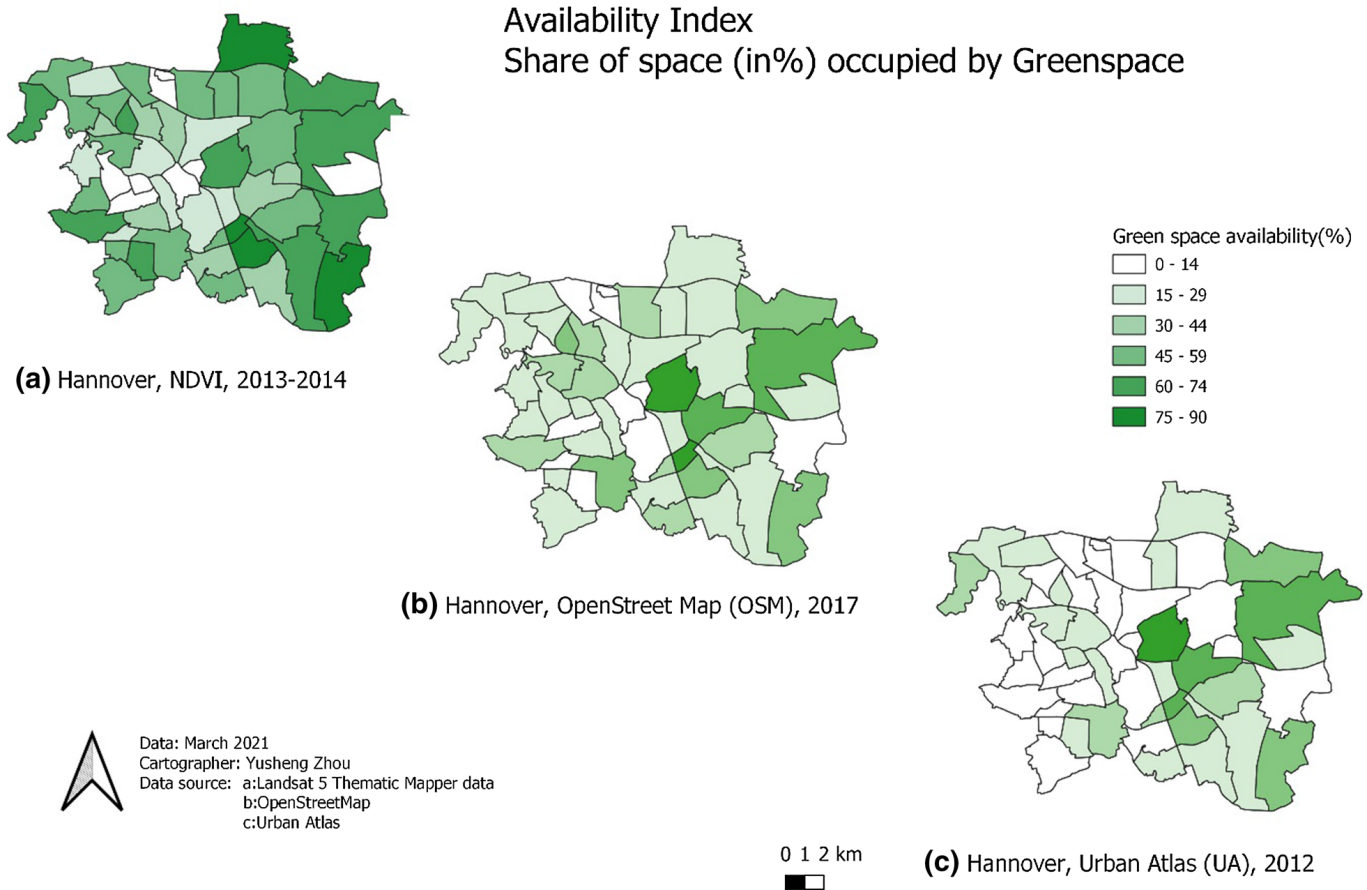
Distribution of greenspace exposure using three different measurements. NDVI: Measurement of calculating the Normalized Difference Vegetation Index (NDVI) by analysing Landsat satellite images

Within the different greenspace measurements, UA and OSM showed strong positive correlations (Fig. 1). Having moderate positive correlations with UA and OSM, the total greenspace area from NDVI should represent the upper limit of greenspace provision. However, NDVI tended to report high levels of greenspace for some areas in which the other two indicators showed higher levels (Fig. 2).

Figure 2 illustrates spatial differences between the greenspace measurements in the city of Hannover. The maps show a high level of agreement between OSM and UA, but the tendency for UA to indicate lower levels of greenspace is evident. At the same time, the availability index of UA and OSM shows that greenspace availability tends to be high in central areas.

Table 3 presents the results of the multilevel regression analysis. Three models were created to compare the effects of greenspace availability according to different data sources. Across all models, children's sex, migration background, number of siblings in the family, birth weight, and parental educational level were all significantly associated with the children's weight status. For the area-level information, the unemployment rate of the area was significant in all models, while residential density was significant for the two land use driven data source models (UA and OSM datasets). Only greenspace availability as defined by NDVI was significantly associated with BMI, meaning that a lower BMI z-score was associated with higher greenspace in Landsat imagery. The other two data sources showed no significant association.

**Table 3:** Estimates from two-level linear modelling predicting pre-school children’s BMI z-score (Data from school entrance examination, city of Hannover, Germany, 2010–2014, n = 22,678)^§^

	Model 1a Availability index by NDVI		Model 1b Availability index by UA		Model 1c Availability index by OSM	
	b (SE)	95% CI	b (SE)	95% CI	b (SE)	95% CI
Individual-level independent factors						
Girls (Ref. Boys)	0.09* (0.01)	(0.06; 0.12)	0.09* (0.01)	(0.06; 0.12)	0.09* (0.01)	(0.06; 0.12)
Children with migration background (Ref. German children)	0.18* (0.02)	(0.15; 0.22)	0.19* (0.02)	(0.16; 0.22)	0.19* (0.02)	(0.15; 0.22)
Single parent/Blended family (Ref. Nuclear family)	0.07 (0.04)	(− 0.01; 0.16)	0.08 (0.04)	(− 0.01; 0.16)	0.08 (0.04)	(− 0.01; 0.16)
Two or more siblings (Ref. one or none siblings)	0.06* (0.02)	(0.02; 0.09)	0.05* (0.02)	(0.02; 0.09)	0.06* (0.02)	(0.02; 0.09)
Childcare less than 3 years (Kita) (Ref. 3 year or longer)	− 0.02 (0.02)	(− 0.06; 0.02)	− 0.02 (0.02)	(− 0.06; 0.02)	− 0.02 (0.02)	(− 0.06; 0.02)
Parental educational level(Ref. Higher education)						
Lower education level	0.27* (0.02)	(0.24; 0.31)	0.27* (0.02)	(0.24; 0.31)	0.27* (0.02)	(0.24; 0.31)
Middle education level	0.15* (0.02)	(0.12; 0.19)	0.15* (0.02)	(0.12; 0.19)	0.15* (0.02)	(0.12; 0.19)
Birth weight (Ref. Normal)						
High (> 4000 g)	0.37* (0.02)	(0.32; 0.42)	0.37* (0.02)	(0.32; 0.41)	0.37* (0.02)	(0.32; 0.42)
Low (< 2500 g)	− 0.25* (0.03)	(− 0.31; − 0.19)	− 0.25* (0.03)	(− 0.31; − 0.19)	− 0.25* (0.03)	(− 0.31; − 0.19)
Area-level socio-demographic factors						
Residential density	0.01 (0.01)	(− 0.02; 0.02)	0.01* (0.01)	(0.001; 0.03)	0.01* (0.01)	(0.001; 0.03)
Unemployment rate (%)	0.04* (0.02)	(0.01; 0.08)	0.05* (0.02)	(0.01; 0.08)	0.04* (0.02)	(0.01; 0.08)
Area-level greenspace indicators						
Greenspace availability index	− 0.01* (0.01)	(− 0.05; − 0.01)	0.01 (0.01)	(− 0.01; 0.03)	0.01 (0.01)	(− 0.01; 0.02)

^*^p < 0.05 (highlighted)

^$^Model 1a: Model on children’s BMI z-score adjusted for individual level factors & other area level factors presented in the table plus greenspace availability by NDVI; Model 1b: Model plus greenspace availability from European Urban Atlas dataset; Model 1c: Model plus greenspace availability from OpenStreetMap dataset

Table 4 presents the results of the interaction terms between parental educational level, sex and children's migration background and green space availability by different data sources. The association between greenspace and body weight was modified by migration background and sex, restricted to greenspace measured by NDVI. In boys and migrant children, higher greenspace availability was significantly associated with a lower BMI compared to girls and non-migrant children.

**Table 4:** Associations between children’s BMI z-score and interaction terms of greenspace availability (Data from school entrance examination, city of Hannover, 2010–2014, n = 22,678) ^$^

	Model 2a		Model 2b		Model 2c	
	(Availability index by -NDVI)		(Availability index by UA)		(Availability index by OSM)	
	β (SE)	95% CI	β (SE)	95% CI	β (SE)	95% CI
Sex * Greenspace Availability	0.03* (0.01)	(0.01, 0.06)	− 0.02 (0.01)	(− 0.04, 0.01)	− 0.01 (0.01)	(− 0.03, 0.02)
Migration background* Greenspace Availability	− 0.001	(− 0.09, − 0.03)	0.01 (0.02)	(− 0.02, 0.04)	− 0.01 (0.02)	(− 0.03, 0.03)
Lower education level * Greenspace Availability	− 0.02 (0.02)	(− 0.06, 0.01)	− 0.02 (0.02)	(− 0.06, 0.13)	− 0.03 (0.02)	(− 0.06, 0.01)
Middle education level * Greenspace Availability	0.01 (0.02)	(− 0.03, 0.04)	− 0.01 (0.02)	(− 0.04, 0.03)	− 0.01 (0.02)	(− 0.04, 0.03)

^$^ Adjusted for sex, migration background, family structure, number of siblings, length of child day care participation, parental educational level, birth weight, residential density, unemployment rate

^*^p < 0.05 (highlighted)

**Fig. 3:**
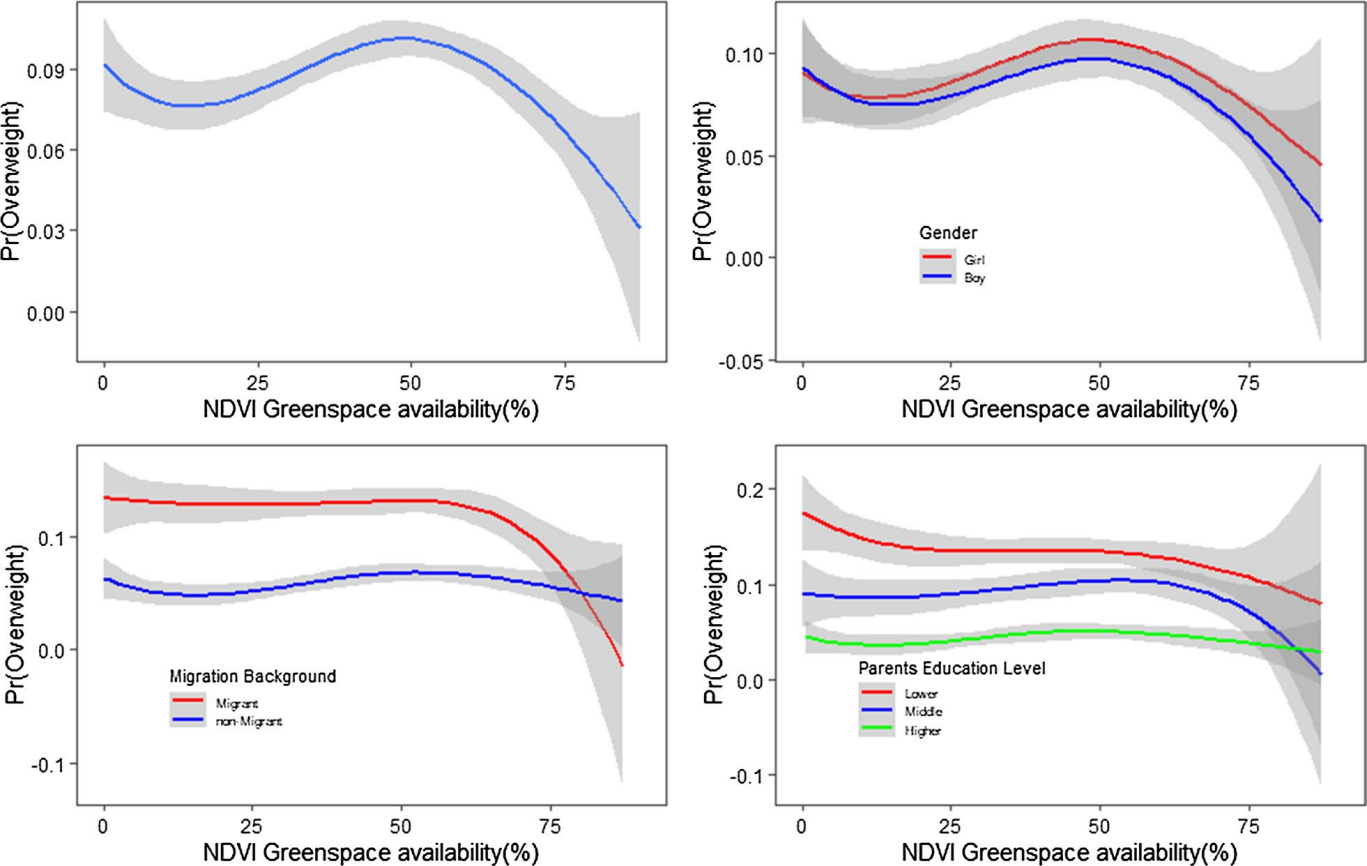
Estimated exposure–response curves for the probability of being overweight for greenspace availability. Estimated exposure–response curves and 95% CIs for the probability of being overweight for greenspace availability (df = 4) for all children (upper left), stratified by gender (upper right), migration background (lower left) and parents education level (lower right). (Y-axis: probability of being overweight (Pr(Overweight)), X-axis: greenspace availability from NDVI. (Data from school entrance examination, city of Hannover, Germany, 2010–2014, n = 22,678)

To further examine these results, spline regression models stratified by sex and migration background were performed. The association of greenspace availability from NDVI with being overweight was significantly different (p < 0.05) from linearity (Fig. 3) in our main model using four degrees of freedom. Similar patterns of the association were found for models with three and five degrees of freedom. From the results of spline models, the exposure–response curves demonstrated patterns with increases and decreases at different levels of greenspace availability (Fig. 3). The downward curve for the exposure–response relationship for higher levels of greenspace is consistent with the results from the BMI z-score interaction term analysis: Greenspace availability tends to show a more negative association with the probability of being overweight for boys and migrant children.

## Discussion

The main goal of this study was to compare results from three different greenspace data sources to determine their relative level of agreement. We used a multilevel modelling approach to analyse associations between greenspace and children's weight status. The degree of surrounding greenspace varied considerably between each of the measurements. The greenspace availability per district area derived by NDVI was on average 42%, by OSM 29% and UA 22%, with OSM and UA being strongly correlated. Only the greenspace availability derived by NDVI was weakly associated with children's weight: The more greenspace availability there was, the lower their BMI. The association was slightly higher for boys and migrant children than for girls and non-migrant children but restricted to the highest levels of greenspace availability.

### Comparison between different data sources

UA and OSM are high-quality and well-validated data sources, while NDVI has been applied in many spatial analyses of greenspaces. Comparisons among these three different data sources clearly showed that NDVI detects surrounding greenspace more comprehensively, presenting the greenspace provision's upper limit among these measurements. The greenspace availability based on UA and OSM is considerably less than that based on NDVI.This difference derives mainly from the fact that UA and OSM were not able to cover private greenspaces, especially in residential areas. Meanwhile, the NDVI derived from Landsat images includes croplands, forests, lakes and marshes, which are not necessarily recreational friendly and not necessarily associated with promoting physical activity in children [28].

Theoretically, these two land use data sources may provide more meaningful information to better distinguish the type of greenspace. However, this type of approach has its own limitations for comparative research due to the mean spatial heterogeneity of land cover across cities [29]. The coarseness of some data introduces the problem of derived indicators. For example, indicators based on count metrics may not be completely accurate, because adjacent spaces may appear as separate spaces in the data but exist as one united space [30]. Besides, the edge effect also appears in the area-level analysis, that is, those who live near a vast space may exhibit outliers with an extremely high level of access, especially those located on the edge of town/city or in the rural area [30]. These two land-use data sources have their own issues. According to OSM data, the contributed features may suffer from a lack of consistency such that they have dissimilar geometrical accuracy and frequently overlap with each other [31]. Simultaneously, although the data were generated from a highly reliable approach, UA data maintain issues such as a lack of categories for private greenspaces. To increase the accuracy of the data, researchers have applied OSM data to assist the validation of land use maps from UA data. According to studies of converting OSM data into a map using UA data by Fonte et al. [32, 33], the accuracy indices validated by OSM data were not significantly different from those obtained by photo interpretation.

For comparison across different data sources, inconsistencies in greenspace definitions are challenging [4, 34]. The greenspace categories in different data sources do not necessarily overlap. This makes it difficult to compare surrounding greenspace obtained from different data sources, not to mention that the detailed categories or types of greenspace are vague in most data sources. For example, none of the included data sources distinguish between the public and private ownership, while some research objectives may be required to distinguish between them. Thus, the study design used here cannot provide a definitive perspective on the choice of data source, but the intriguing result merits further investigation.

### Association between greenspace and weight status

The built environment in which children live, play and eat provides an important context that may influence overweight risk [35]. Research has shown that publicly provided recreational infrastructure is positively associated with children's physical activity [36, 37]. This indicates that children and their parents tend to be more physically active in a conducive environment [37]. Mechanistically, exposure to greenspace may promote health-related behaviours, mitigate harmful environmental exposures to air pollution, heat, and noise, and encourage stress alleviation [34, 38]. All these beneficial effects of greenspace are involved with the pathophysiologic pathways affecting children's independent mobility and willingness to engage in physical activity behaviour, which eventually lead to changes in weight [34, 39].

Our previous analysis on the association between area-level greenspace and children’s overweight using data from the OSM dataset failed to show a significant association with the BMI z-score [40]. Given the results from this study, this might be due to the data source to measure greenspace exposure. The results from other studies support this hypothesis: a study from New Zealand based on national administrative records discovered that greenspace was not significantly related to children’s overweight [12], and a study from Berlin, Germany [41] using a land-use categorical database also showed a nonsignificant association among children. A review of German studies of built environment effects on health concluded that there was no association between greenspace and obesity [42]. In contrast, studies using NDVI data [43–45] often found that higher surrounding greenspace levels were associated with lower BMI z-scores suggesting that the choice of data source of greenspace has relevant implications on its association with health outcomes.

As NDVI is sensitive to both public and private greenspace, the significant association between children’s overweight and greenspace might raise the possibility that it is the private greenspace that holds a specific influence on children’s development [46]. Case studies demonstrated that privately owned greenspaces (e.g. backyards or domestic gardens) often acted as a substitute for public greenspaces (e.g. communal gardens or neighbourhood parks) in residential areas [47]. If public greenspace exerted greater influence, we would have expected to see similar associations in UA and OSM. However, the mere presence of greenspace does not necessarily mean its use will provide its effect. The major limitation of applying NDVI in epidemiological studies is its inability to distinguish between different types of greenspaces which may promote physical exercise to varying degrees due to characteristics such as size and available facilities [48]. Simultaneously, the greenest areas in an urban setting (e.g. urban forests) might relate to having fewer transport destinations within walking distance, which leads to greater car dependency [49]. Therefore, further study should consider that the different types of greenspace may have different effects on promoting physical activity and hence different protective influences on children’s weight gain.

### Effect of greenspace may differ in subgroups

Greenspace enables outdoor physical activity which may be an important preventive factor for children’s overweight. However, research evidence suggests that the influence of individual characteristics is greater than the influence of built environmental determinants on the level of physical activity [50]. Individual socioeconomic status may modify correlations between greenspace availability and the willingness to engage in physical activity [2, 14]. Correspondingly, Lovasi et al. found a weak association between greenspace and the physical activity rate, especially in low-income communities [8]. Another reason might be safety aspects, as research from North America points out that areas with high-density trees may have higher risk of crime, which leads to a lower willingness to engage in physical activity due to safety considerations [51].

Our study noted that the association between greenspace and body weight could be modified by sex and migration background, which is consistent with several greenspace-related research findings in adulthood. The evidence for sex is mixed and restricted to adults. Some studies indicate a tendency for a more substantial effect in women [1, 52–54], while others have shown no associations [55]. Several studies considered race/ethnicity effects for pregnant woman [56, 57] and school children [28]. They note that the potential built environment reflecting cultural preferences has plausible implications for health behaviour and outcomes [58]. Therefore, further research on children should include more comprehensive and multidimensional metrics.

### Limitations

Greenspace availability is not the only indicator to measure greenspace. Generally, access to greenspace is often used in epidemiological studies as well [13, 14, 59]. It can be estimated by multiple indicators, including the Euclidean distance to a park, the network distance to a greenspace area, and the presence of park within a given area around the home address [14]. Furthermore, some studies explored the perception of greenspace on willingness to use greenspace facilities and actual use by questionnaires [37, 39, 60]. The absence of a standard indicator representing greenspace has become an important barrier to the generalization of the study results.

This study was limited to analysing greenspace at the area level. This is partly due to the unavailability of individual home address information. Additional dimensions of indicators on a better scale (i.e., zip code and home address) would greatly enhance the precision of exposure measurement [14]. When performing spatial analysis, defining built environment values is undermined by being dependent on spatial scales and spatial units. This phenomenon is described by the modifiable areal unit problem (MAUP) [61]. The MAUP is an issue in spatial analysis for studies in geography. It describes that built environment values may vary with the spatial scale for which data are available and the boundaries between spatial units [62]. For now, the MAUP is still rarely addressed in practice. A systematic review in 2014 reports that MAUP was recognized in only 1% of papers using spatially aggregated data [61]. A potential way to mitigate the impact of the MAUP is to create a geographical structure with high heterogeneity zoning units. Other studies proposed to conduct a sensitivity analysis [63]. Therefore, increasing awareness of this methodologically relevant issue is critically needed in health geography analysis.

This study has other limitations in the method design. First, focusing on a sample of one city was informative with respect to the accuracy of greenspace measurements achieved, but had reduced generalizability. Greenspaces in our target, the city of Hannover, constitute 11.4 percent of the total surface area, earning it the title of Germany's greenest city. This study may not cover the entire situation of the built environment in German cites, and the results might be different for other cities and countries. Second, the OSM data used were from a slightly later period (2017) than the other two data sources (approximately 2012–2013); it is possible that differences in the greenspace detected were due to changes over time. As the presence of greenspace in Germany is relatively persistent over these ten years, the bias resulting from this measurement error is likely to be minor [29]. Third, our results cannot prove any causal relationship between greenspace exposure and children’s overweight according to the cross-sectional study. Associations may be prone to residential self-selection, as families are likely to select their neighbourhood according to their personal preferences, lifestyle, and culture, and consequently are likely to prefer healthier, greener neighbourhoods [40, 64, 65]. Residual confounding cannot be excluded, as the range of confounders adjusted for in this study was limited and did not include potential risk factors for being overweight such as eating habits and actual physical activity. Regarding the outcome measure, BMI percentiles and z-scores have the potential to misclassify health-related outcomes in children. Previous studies have evaluated the validity of BMI z-score predicting overweight in children and conclude that it is a weak predictor of the changes in total body fat [66]. However, due to ease of acquisition, BMI standardized for age and sex is still the most widely used clinical outcome variable. The addition of other anthropometric measurements such as waist circumference and skinfolds measurements to BMI z-score assessment may help to identify those children with excess body fat.

## Conclusion

Surrounding greenspace measured by NDVI reaches nearly twice the amount of the greenspace proportion of OSM and UA, identifying NDVI as the more comprehensive data source. Negative associations with BMI were only detected for NDVI-based greenspace availability, suggesting that data sources based on land use categories, such as UA and OSM, are less suitable to reflect the surrounding greenspace that is relevant for health outcomes. Ultimately, achieving accurate and standardized surrounding greenspace measurements can be difficult, especially considering individual characteristics and data availability. It is beyond the scope of this article to recommend the most appropriate greenspace data source. However, researchers should consider and transparently report the impact of different greenspace data sources on the relationship between surrounding greenspace and health outcomes.

## Data Availability

All data generated or analyzed during this study are available from the corresponding author on reasonable request.

## Abbreviations

NDVI: The Normalized Difference Vegetation Index
OSM: The OpenStreetMap dataset
UA: The European Urban Atlas dataset
BMI: The body mass index
SES: Socioeconomic status
